# Using simulated wildland fire to assess microbial survival at multiple depths from biocrust and bare soils

**DOI:** 10.3389/fmicb.2023.1123790

**Published:** 2023-03-17

**Authors:** Brianne Palmer, Nicole Pietrasiak, Polina Cobb, David Lipson

**Affiliations:** ^1^Department of Biology, San Diego State University, San Diego, CA, United States; ^2^Department of Plant Science, University of California, Davis, Davis, CA, United States; ^3^School of Life Sciences, University of Nevada, Las Vegas, Las Vegas, NV, United States; ^4^Department of Integrative Structural and Computational Biology, Scripps Research Institute, San Diego, CA, United States

**Keywords:** biological soil crust, fire, cyanobacteria, resistance, resilience

## Abstract

**Introduction:**

Surface soil microbial communities are directly exposed to the heat from wildland fires. Due to this, the microbial community composition may be stratified within the soil profile with more heat tolerant microbes near the surface and less heat tolerant microbes, or mobile species found deeper in the soil. Biological soil crusts, biocrusts, are found on the soil surface and contain a diverse microbial community that is directly exposed to the heat from wildland fires.

**Methods:**

Here, we used a simulated fire mesocosm along with a culture-based approach and molecular characterization of microbial isolates to understand the stratification of biocrust and bare soil microbes after low severity (450°C) and high severity (600°C) fires. We cultured and sequenced microbial isolates from 2 to 6 cm depth from both fire types.

**Results:**

The isolates were stratified along the soil depth. Green algal isolates were less thermotolerant and found in the deeper depths (4–6 cm) and the control soils, while several cyanobacteria in Oscillatoriales, Synechococcales, and Nostocales were found at 2–3 cm depth for both fire temperatures. An Alphaproteobacteria isolate was common across several depths, both fire types, and both fire temperatures. Furthermore, we used RNA sequencing at three depths after the high severity fire and one control to determine what microbial community is active following a fire. The community was dominated by Gammaproteobacteria, however some Cyanobacteria ASVs were also present.

**Discussion:**

Here we show evidence of stratification of soil and biocrust microbes after a fire and provide evidence that these microbes are able to survive the heat from the fire by living just below the soil surface. This is a steppingstone for future work on the mechanisms of microbial survival after fire and the role of soil insulation in creating resilient communities.

## 1. Introduction

The recovery of microorganisms after a disturbance is a key component of ecosystem restoration (Harris, 2003). There are several theories and experiments that attempt to explain microbial recovery through resistance or resilience. A resistant microbial community is insensitive to disturbance while a resilient community may be altered but eventually return to the original state (Shade et al., 2012). How microbes respond depends on if the disturbance is a press disturbance that occurs over long time scales (i.e., climate change) or a pulse disturbance which is s short-term disruption (i.e., fire) (Shade et al., 2012).

Fire, as pulse disturbance, can alter the microbial community in a variety of ways. Fire can reduce microbial biomass (Dooley and Treseder, 2012; Fontúrbel et al., 2012) and is mediated by the severity of the fire (Dooley and Treseder, 2012). Low severity fires do not alter microbial chemistry but high severity fires do (Pourreza et al., 2014). Sometimes, fire may only have a small effect on soil functioning if the insulating effect of the soil matrix is maintained keeping soil temperatures during a fire at relatively low temperatures (Fontúrbel et al., 2012). Microbial communities on the soil surface (0–5 cm) are most impacted by fire, with recorded decreases in microbial diversity in moderate and high severity wildfires compared to low severity wildfires in a forest ecosystem (Nelson et al., 2022).

Fire severity describes the interactions between the fire temperatures, duration, and other biotic and abiotic factors (Neary et al., 1999). In a simplified model, a high severity fire would have a high temperature and a long duration, and a low severity fire would have a relatively low temperature and short duration. For microbes, the temperature of the soil and the amount of time that the soil is exposed to heat may determine the survival of microorganisms (Hart et al., 2005). The duration is determined by the type and the amount of fuel (Neary et al., 1999). In grasslands and shrublands, heat is transferred into the soil primarily through radiation and convection and is dependent on the water content, structure, and organic matter in the soil (Neary et al., 1999). For example, a grassland fire may have a high release of heat but burn so quickly that much of the initial heat fails to penetrate deep into the soil depths (Neary et al., 1999). In the shrub-dominant chaparral ecosystem, flames can linger longer on the shrub islands and peak fire temperatures on the soil surface range from approximately 250 to 700°C (DeBano et al., 1979). However, at 2.5 cm depth, temperatures ranged from approximately 70 to 280°C (DeBano et al., 1979), well within the temperature range of thermophilic microorganisms (Brock, 2012). The development of a heat-stratified soil profile where temperatures drop precipitously as depth increases (Campbell et al., 1994; Massman, 2012), suggests there may also be stratified mortality, with increased survival at greater depths, although survival is highly dependent on the type of organism (Pingree and Kobziar, 2019). Soil microbial communities can be relatively resistant to fire (Pressler et al., 2019). In some cases, microbes can enter a dormant state during the fire and be reactivated after the heat from the fire dissipates (Lennon and Jones, 2011; Wang et al., 2015).

Studying the effect of these fire processes on soil microbial communities is challenging and has been addressed in several studies [reviewed in Dove and Hart (2017) and Pressler et al. (2019)] but these types of studies often use space-for-time replacements, comparing burned and unburned plots which allows for little room for manipulation to explore fire parameters. As a result of this gap in experimental fire and microbial community studies, Bruns et al. (2020) developed a “pyrocosm” system to experimentally test the effects of fire on a microbial community in a controlled setting (Bruns et al., 2020).

Here, we use our own form of pyrocosm with Sanger and RNA sequencing to evaluate the effect of fire on a particular soil microbial community – biological soil crusts (biocrusts). Biocrusts are composed of communities of microscopic and macroscopic organisms living on the soil surface and cover about 12% of the soil surface globally (Rodríguez-Caballero et al., 2018). They are important for a variety of ecosystem functions including nutrient cycling (Belnap, 2002; Elbert et al., 2012), water retention (Eldridge et al., 2020), and soil stability (Rodríguez-Caballero et al., 2012). Fire can have varying impacts on biocrusts from altering their cover (Palmer et al., 2020), changing the photosynthetic biomass (Johansen, 2001), and changing the microbial community (Aanderud et al., 2019). As biocrust microbes, by definition, reside at the soil surface and are at immediate risk of mortality by high-temperature fires on the soil surface while microbes in the deeper layers may survive (DeBano et al., 1979; Hart et al., 2005; Cairney and Bastias, 2007).

In a California coastal grassland, we previously found that the biocrust microbial community is unaltered 1 year after a fire (Palmer et al., 2022). This suggests that biocrusts may have the potential to swiftly recover from a fire. Therefore, using our pyrocosm system, we sought to understand the depth of mortality of biocrust microorganisms after low and high temperature fires using culturing combined with Sanger and RNA sequencing.

## 2. Methods

### 2.1. Biocrust collection

In June 2020, we collected twenty 10 cm deep cores of intact biocrusts and twenty cores of bare soil from the coastal sage scrub habitat at the Santa Margarita Ecological Reserve (SMER) (33.44128 N, −117.1644 W) in Riverside Country, California, USA. The biocrusts are classified as lichen and cyanobacteria dominant and comprise much of the open space at the reserve. The bare soil and the soil below the biocrust cores are classified as a rocky loam Alfisol collected from SMER (Soil Survey Staff, 2021). Soil cores with and without biocrusts were stored in 15 cm silicone cups at room temperature before burning.

### 2.2. Fire simulation

Using the Campbell et al. (1994, 1995) soil heat transfer model, which includes a variable for soil moistures, we developed a model to understand the expected heat stratification outcomes from the simulated fire (Campbell et al., 1994, 1995). Additionally, we used the equations described by Mercer and Weber (2001) to create a right-skewed curve as is typical during a fire (Mercer and Weber, 2001). We calculated the bulk density and the volumetric water content from the SMER soils and used the soil organic matter, mineral soil by volume, and organic matter by volume measurements from previous work at SMER (Pérez Castro et al., 2019). The thermal conductivity (gamma) was set to 0.03bW/cm*K based on estimates from Ochsner et al. (2001). The initial temperature was set to 20°C for both models. The low-intensity fire had a maximum temperature of 450°C, and the high-intensity fire had a maximum temperature of 600°C. These values were based on soil temperature estimates in chaparral (DeBano et al., 1979). For each fire type, it took 600 s to reach the maximum temperature then stayed at the maximum temperature for 60 s. There was an 1800 s decay period as it returned to the basal temperature. The result was a model of the temperature fluctuations every second for every 0.1 cm depth.

**Figure 1:**
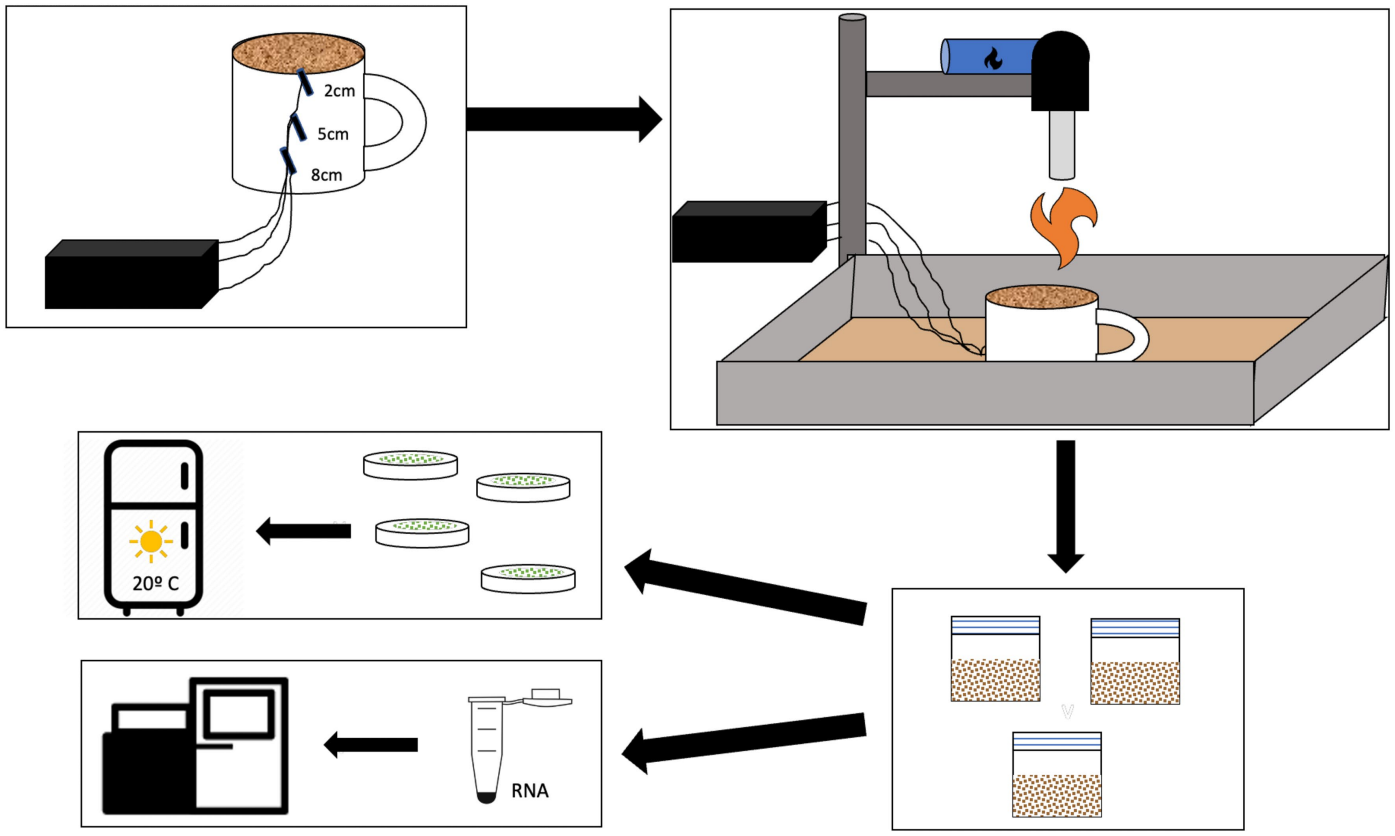
Schematic of the burn methods and downstream analyses. Thermocouples were placed at 2, 5, and 8 cm depth in a ceramic mug which was then placed in a tray of sand below a calibrated blow torch. After burning the soil and biocrusts in the mug, each cm was separated into separate sterile bags. We used soil from each depth to inoculate BG-11 agar plates and allowed the microbes to grow in a growth chamber. A subset of the samples was used for RNA extraction and sequencing.

The fire simulation within the pyrocosm was performed using a blow torch in a controlled laboratory setting. This ensures control over the soil temperature. Previous studies have used this method to test the effects of fire on small scales (Hean and Ward, 2012; Bruns et al., 2020). A blowtorch may more closely model a wildland fire than a similar temperature in the muffler furnace due to the difference in heat conduction. Before the burn, the samples were moved to ceramic mugs. Each mug had a small hole in the bottom where we inserted thermocouples at 8 cm, 5 cm, and 2 cm depth before transferring the soil from the silicon cups used in the field. The thermocouples were used to track the temperature of the soil at three different depths and the values were compared to the model we developed. We compared the temperatures between each treatment using a t-test. The surface of the soil was monitored using a laser thermometer pointed at the hottest area of the soil, directly under the blowtorch flame. The blowtorch was attached to a stand, and we calibrated the height of the stand for each treatment to ensure consistency between samples (Figure 1). The mug was placed in a tray full of sand to allow the heat to diffuse from the bottom of the mug. For each sample, we recorded the ramp-up time and ensured it stayed at the maximum temperature (450°C or 600°C) for 60 s before turning off the blowtorch and letting the mugs cool for 10 min. This cooling period allows the heat from the surface to dissipate into the deeper soil layers, similar to wildland fire, and was accounted for in our model. After the cool-down period, we separated the soil into each cm depth by using a sterile spoon and ruler to scoop out each cm and placed it in a sterile plastic bag. The spoon and ruler were sterilized with 95% ethanol between every centimeter. In total, we had six treatments: low-temperature biocrust, high-temperature biocrust, low-temperature bare soil, high-temperature bare soil, biocrust control, and bare soil control and soil samples were taken at each cm depth (1–10 cm) for culturing and at four depths (1, 2, 5, and 8 cm) for RNA extraction (See sections on Culturing and RNA sequencing). Each treatment was replicated 6 times. After sampling, each plastic bag containing 1 cm of soil was stored at 4°C, although microbes are active at this temperature, we sought to provide an environment where the microbial community could grow and begin to recover from the fire.

### 2.3. Culturing, DNA extraction, and PCR

After the burn, we cultured each sample (*n* = 360) on individual Petri-dishes with BG-11 agar medium. The composition of the media is described in (Stanier et al. (1971)). We used BG-11 media to select for cyanobacteria and other photosynthetic microorganisms that are the building blocks of biocrust (Büdel et al., 2016). For each sample, we created a soil slurry using 1 gram of soil from each depth which was added to 10 ml of distilled water and diluted it to 10^−2^. This dilution was streaked onto BG-11 plates and placed in an incubator at 25°C with a radiance of 20.3 μmol m^−2^ s^−1^, and with 12 h of light and 12 h of darkness. Every week, we recorded the presence or absence of colony-forming units on the plates. After three months, there was enough growth on the plates to begin DNA extractions. Differences in the presence or absence of colonies on the plates between soil type (biocrust versus bare) and temperature was analyzed using a chi-squared test.

All plates that had CFUs (colony forming units), were used to isolate single colonies which were transferred to new Petri-dishes with BG-11 made with low-temp melting agar. This is because of the often-filamentous nature of biocrust organisms. The cyanobacteria often grow into the agar, and it can be difficult to remove the culture for DNA extraction. After growing on the low-melt agar for one month, we began DNA extraction using unialgal cultures.

For each sample, we scraped the biomass and agar off the plate with a sterile loop and placed it into a 1.5 ml collection tube. We then added 1 ml of sterile water that was warmed to 60°C to help melt the agar. The collection tubes were centrifuged for 5 min at 13,000 rpm, incubated for 30 min at 65°C, then centrifuged again for 5 min. Incubation and centrifugation were repeated until the agar was completely melted. After the agar was melted, we removed the supernatant, leaving the pellet of biomass, and continued with DNA extraction. We then used the phenol-chloroform DNA extraction method. We placed each sample in a 1.5 ml collection tube and filled it with 100 mg of UV sterilized beads. Then, we added 500 μl of lysis buffer (TE with SDS) to each sample and vortexed for 10 min. We then added 5 μl proteinase-k and incubated the samples at 37°C for 30 min. Next, we added 25 μl 0.5 M NaCl, 20 μl 0.5 M CTAB NaCl, and 500 μl chloroform. The samples were vortexed and centrifuged at 13,000 rpm for 5 min. Then, we added 500 μl of phenol-chloroform, vortexed, and centrifuged for 5 min. We moved the aqueous solution into sterile collection tubes and precipitated it with 0.7 volumes of 100% isopropanol. After 30 min, we rinsed with 500 μl cold ethanol and centrifuged at 13,000 rpm for 10 min. We removed the supernatant and let the pellet air dry in the fume hood for 24 h. We resuspended the DNA pellet in 100 μl Tris buffer.

Because there are a variety of photosynthetic organisms found in biocrust, we used both prokaryotic (16S) and eukaryotic (ITS) primers. The DNA was amplified using PCR. For a solution of 25 μl, we used 2.5 μl 10x Taq polymerase buffer, 2.5 μl dNTP, 1.5 mM MgCl_2_, 1.25 μl forward primer, 1.25 μl reverse primer, 0.2 μl Taq-polymerase, 1 μl BSA, 12.8 μl DNA-free water, and 2 μl template. The PCR settings included an initial denaturation at 94°C for 3 min and the following cycle repeated thirty times: denaturation at 94°C for 30 s, annealing at 55°C for 30 s, elongation at 72°C for 60 s, then a final elongation at 75°C for 6 min and cooled to 4°C. The quality of the PCR product was assessed using gel electrophoresis, only samples with a clear band were sequenced. In total, we extracted DNA and performed PCR on 80 colonies spanning the range of depths for each treatment. PCR bands were visible on 25 samples using the 16 s rRNA primers and 22 samples using the ITS primers. Samples with visible PCR bands were used for DNA sequencing. In total, six bacteria cultures were sequenced using f8-27 (5′-AGAGTTTGATCCTGGCTCAG-3′) and r1510 (5′-GGTTACCTTGTTACGACTT-3′. Seventeen bacteria cultures were sequenced using more specific cyanobacteria primers cyanoF (5′ – GGGGAATTTTCCGCAATGGG −3′) and r1510cy (5′ – ACGGCTACCTTGTTACGACTT −3′). One algae sample was sequenced using algSSU1 (5′ -TGGTGGACTCTGCCAGTAG −3′) and algSSU2 (5′ -TGATCCTCCCGCAGGTCCAC -3′). Since only one sample was successfully sequenced with the SSU primers, we tried a separate ITS primer to enhance our sequencing success of the eukaryotic algae. A further nine algae samples were sequenced using ITS1F (5′ – TCCGTAGGTGAACCTGCGG −3′) and ITS4 (5´-TCCTCCGCTTATTGATATGC-3′) (Wilmotte et al., 1993; Nübel et al., 1997; Pryce et al., 2003; Lipson and Schmidt, 2004). All samples were sequenced with Sanger Sequencing technology at Eton Biotech (San Diego, CA). We focused the DNA extractions and sequencing to samples from 1-6 cm depths in all treatments.

It should be noted that this culture-dependent technique does not accurately describe the temperature range of microbial isolates, nor does it reflect the diversity of the microbial community within the samples. It does, however, provide evidence about if biocrust phototrophic microorganisms can survive and grow after a fire at each depth.

### 2.4. RNA extractions and sequencing

We also used the burned soil samples for RNA analyses. We chose RNA extraction over DNA extraction because RNA indicates what organisms are metabolically active rather than dormant after a fire. We extracted RNA from soil and biocrust samples at the same depth of the thermocouples and from the soil surface (1 cm, 2 cm, 5 cm, and 8 cm). We placed 2 g of the sample into a sterile petri-dish, sprinkled it with DI water, and placed it in the 12-h day-night cycle incubator at 20°C for four days. This simulated the environment the soils might experience with a precipitation event after a fire and stimulated the microbial community. After four days, we immediately froze the samples in an ethanol bath with dry ice. Samples were stored at −80°C until RNA extraction.

We extracted the RNA using the Qiagen RNeasy RNA extraction kit (Qiagen, Hilden, Germany) per the manufacturer’s instructions. We then reverse transcribed the RNA using the Quantitect Reverse Transcription kit. Then, we performed a PCR on the cDNA using the Pro305F (5′ – CCTACGGGNBGCASCAG -3′) and Pro805R primers (5′ – GACTACNVGGGTATCTAATCC -3′) with the same settings as the previous PCRs, though the annealing temperature was raised to 56°C, as it was specified for the primers. The samples that were amplified using PCR were then purified using QIAquick PCR Purification Kit (Qiagen, Hilden, Germany). Samples that were still below 20 ng/μl of DNA were further concentrated using a SpeedVac. Purified samples were sequenced at GeneWiz (La Jolla, California, USA). Although we were able to extract RNA from 10 samples, only samples with >20 ng of DNA after RNA extractions, PCR, SpeedVac concentration, and PCR purification were used for subsequent sequencing due to constraints of sequencing samples with low RNA yield for a total of 4 samples comprising the following treatments: biocrust burned at 600°C at 1, 2, and 8 cm and a control sample from 5 cm deep.

### 2.5. Phylogenetic trees

First, Sanger sequenced data were compared with published sequences in GenBank using BLASTn for an initial comparison and to identify which cyanobacterial order sequenced organisms would most likely classify (McGinnis and Madden, 2004). The 16S and ITS sequences obtained from the culture-dependent sequencing were then placed into alignments in preparation for phylogenetic analysis. In addition to our sequences, we added closely related reference sequences as well as sequences from organisms known to be abundant in soils and biocrusts. The sequences were aligned in Mega 11 using ClustalW (Thompson et al., 1994; Tamura et al., 2021). We used the maximum-likelihood tree from IQ tree with 1,000 bootstraps and the default settings (Trifinopoulos et al., 2016). We separated the cyanobacteria into three different trees, one for each represented order – Synechococcales, Oscillatoriales, and Nostocales. For the Cyanobacteria, the trees were rooted with *Gloebacter violaceus* (NR074282). We included one tree for Alphaproteobacteria with *Rhizobium lentis* (NR_137243) as the root and one tree for Chlamydomonas with *Chlorella variabilis* (NW_00543849) as the root. The accession numbers used for each tree are listed in Supplementary Table 1.

### 2.6. RNA analyses

Four samples were used for RNA extraction from biocrust samples burned at 600° at 1 cm, 2 cm, and 8 cm with a control sample at 5 cm depth. We recognize that there are no replicates within this sampling scheme, rather, we included this analysis as an exploratory experiment to test the survival of biocrust RNA during a simulated fire. Raw fastq files were filtered and trimmed to remove the primers, dereplicated, demultiplexed, and the chimeras were removed using DADA2 (Callahan et al., 2016). The samples were classified using both the Silva 138 and CyanoSeq 1.0 databases (Quast et al., 2013; Lefler and Berthold, 2022). We extracted ASVs that were classified as “Cyanobacteriota” from the CyanoSeq database and placed them within a phylogenetic tree with our Sanger sequences and representative sequences from Synechococcales, Oscillatoriales, and Nostocales using IQ tree with 1,000 bootstraps. We also created a tree with eukaryotic algae and the ASVs from CyanoSeq since the phylum “Cyanobacteriota” contains both prokaryotic and eukaryotic organisms. The algae tree included terrestrial algae sequences from NCBI, using the accession numbers from Lewis and Lewis (2005) as a reference (Lewis and Lewis, 2005) as well as our algal Sanger sequences.

## 3. Results

### 3.1. Thermocouple measurements and model

**Figure 2:**
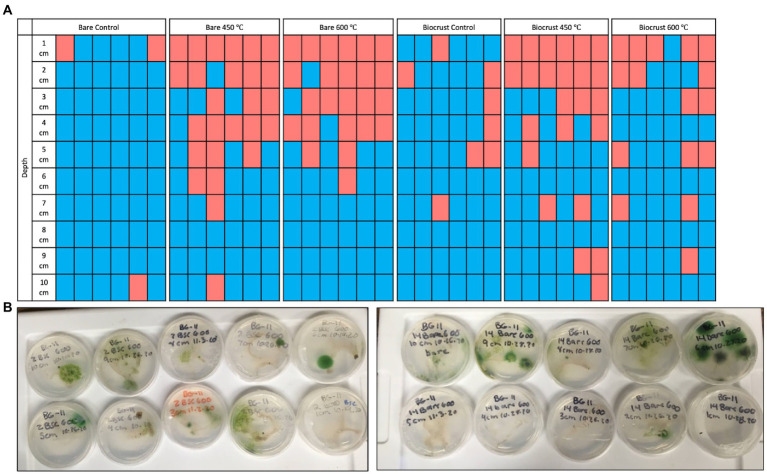
**(A)** The presence/absence of cultures on BG-11 agar plates for each sample at each depth (1–10 cm). Red rectangles indicate there was no growth after 3 months, and blue rectangles indicate there was >1 CFU on the plate after 3 months. **(B)** Photo of ten culture plates from 1 to 10 cm depth for biocrust and bare soil burned at 600°C.

The thermocouples were placed at 2, 5, and 8 cm depths which we compared with the values from the model. Overall, our model captured the comprehensive shape of the temperature curve but was 1 cm off for biocrust. The maximum temperature during the experiment occurred in the bare 600°C treatment at 502. 1°C (Table 1; Figure 2). We compared the average temperatures at each depth during the first 30 min of the experiment which included the ramp up time, ten minutes of maximum heat burn time, and a cool down period and found that the biocrust treatments had consistently lower temperatures at each soil depth (Table 1).

**Table 1:** Results from the *t*-test comparing the temperatures at each depth between biocrust and bare soil recorded by the thermocouples for the first 30 min of the experiment which included the ramp-up, ten-minute burn at the treatment temperature, and a cooldown period.

Fire Temp. (°C)	Depth (cm)	*t*-statistic	Value of *p*	Bare mean (°C)	BSC mean (°C)	Bare Max (°C)	BSC Max (°C)
450	2	6.44	<0.001	123.14	94.80	304. 4	301.7
450	5	12.69	<0.001	53.91	36.88	106.2	68.93
450	8	11.89	<0.001	30.28	23.30	48.52	32.37
600	2	12.85	<0.001	190.95	94.07	502.1	479.3
600	5	13.17	<0.001	46.76	31.36	85.1	51.95
600	8	14.12	<0.001	38.37	23.55	75.86	29.89

### 3.2. Microbial survival

**Figure 3:**
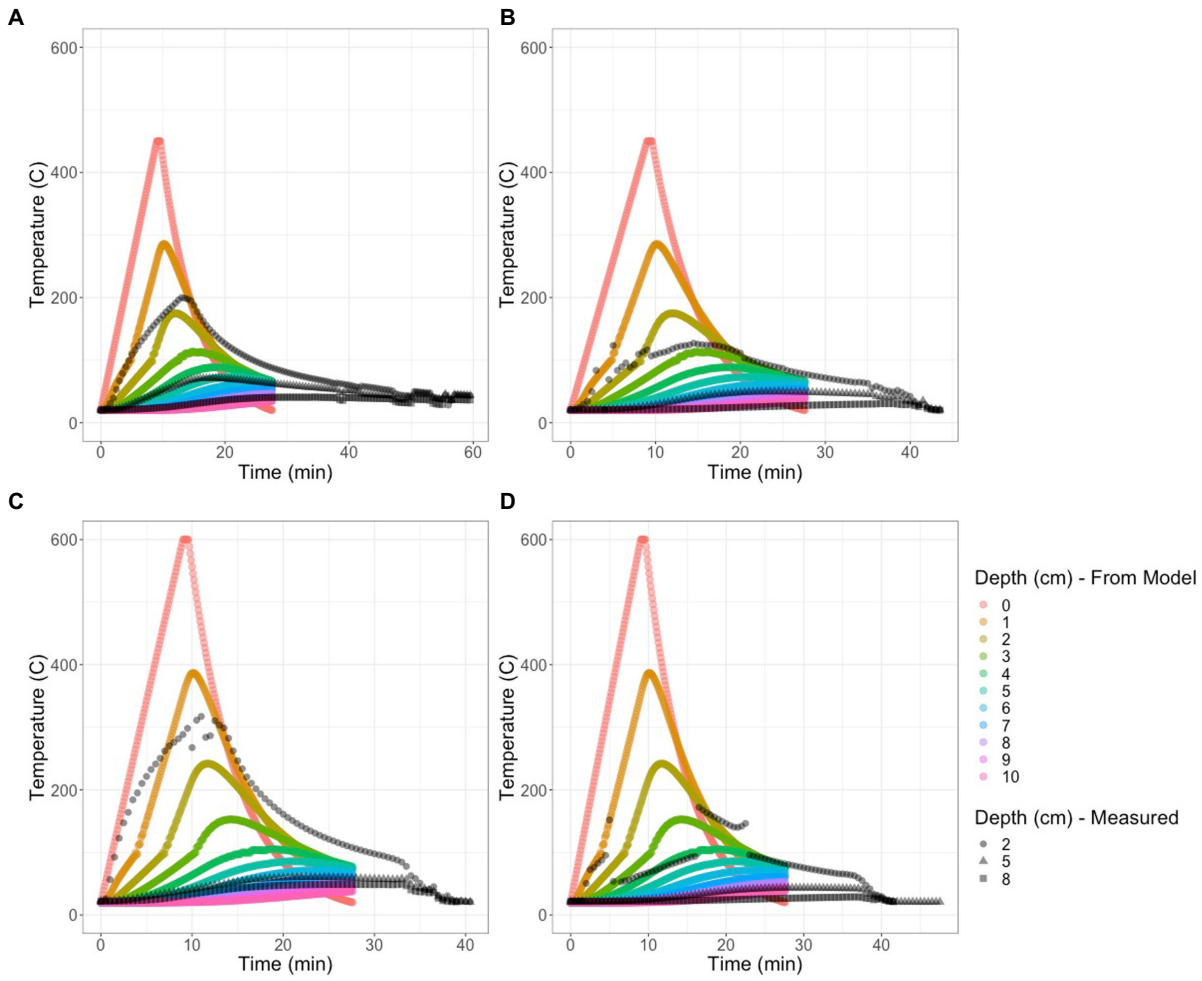
The model predictions of soil temperature for **(A)** bare soil burned at 450°C; **(B)** biocrust burned at 450°C **(C)** Bare soil burned at 600°C, and **(D)** biocrust burned at 600°C. Gaps in the thermocouple data occurred when the thermocouples overheated, particularly at the deeper depths. The actual depth of the thermocouples is assumed to be 2, 5, and 8 cm but they may have shifted during the experiment. Surface temperatures remained consistent throughout with both fire treatments reaching 450 and 600°C, respectively.

Three months after the simulated burns, CFUs were growing on 72% of the plates (n = 259) (Figure 3). We performed a chi-squared test to determine differences in the presence or absence of CFUs for each temperature and cover type (bare and biocrust). There was a significant difference between temperatures (*p* < 0.001) and no difference between cover types (*p* = 0.64). For the bare samples, after the 450°C and 600°C burns, there was growth from 2-10 cm and there was growth on every cm for the control. For the biocrust samples, the 450°C had growth from 3-10 cm and the 600°C burn, and controls had growth from 1-10 cm. One month later, there was one CFU on one plate burned at the top cm at 450°C.

**Figure 4:**
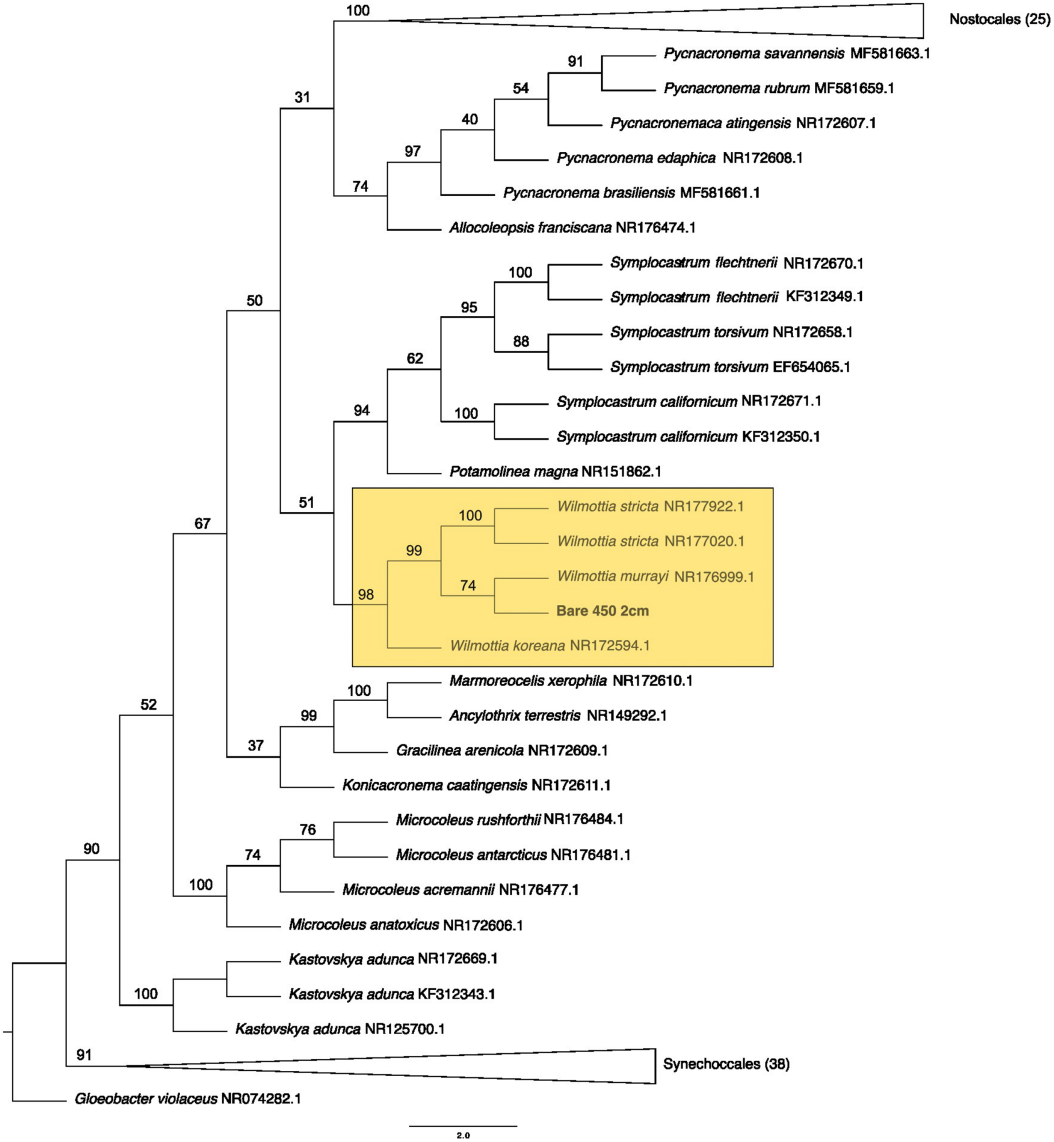
Maximum likelihood tree for Oscillatoriales. Bootstrap values are denoted on the branches. Bootstrap values indicate the percent of the trees with that particular branch placement. Bootstrap values >0.70 are considered well supported.

**Figure 5:**
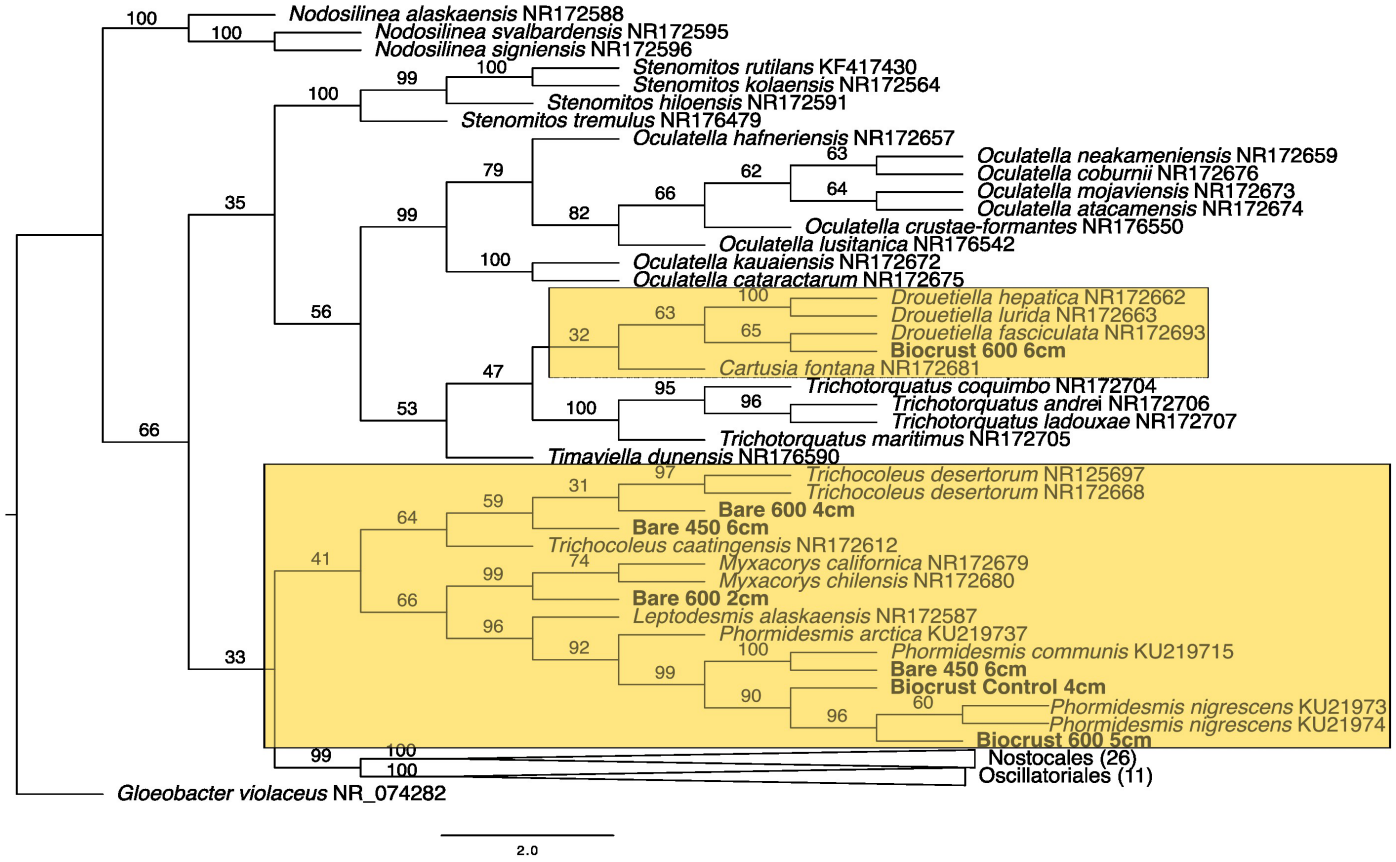
Maximum likelihood tree for Synechococcales. Bootstrap values are denoted on the branches. Bootstrap values indicate the percent of the trees with that particular branch placement. Bootstrap values >0.70 are considered well supported.

**Figure 6:**
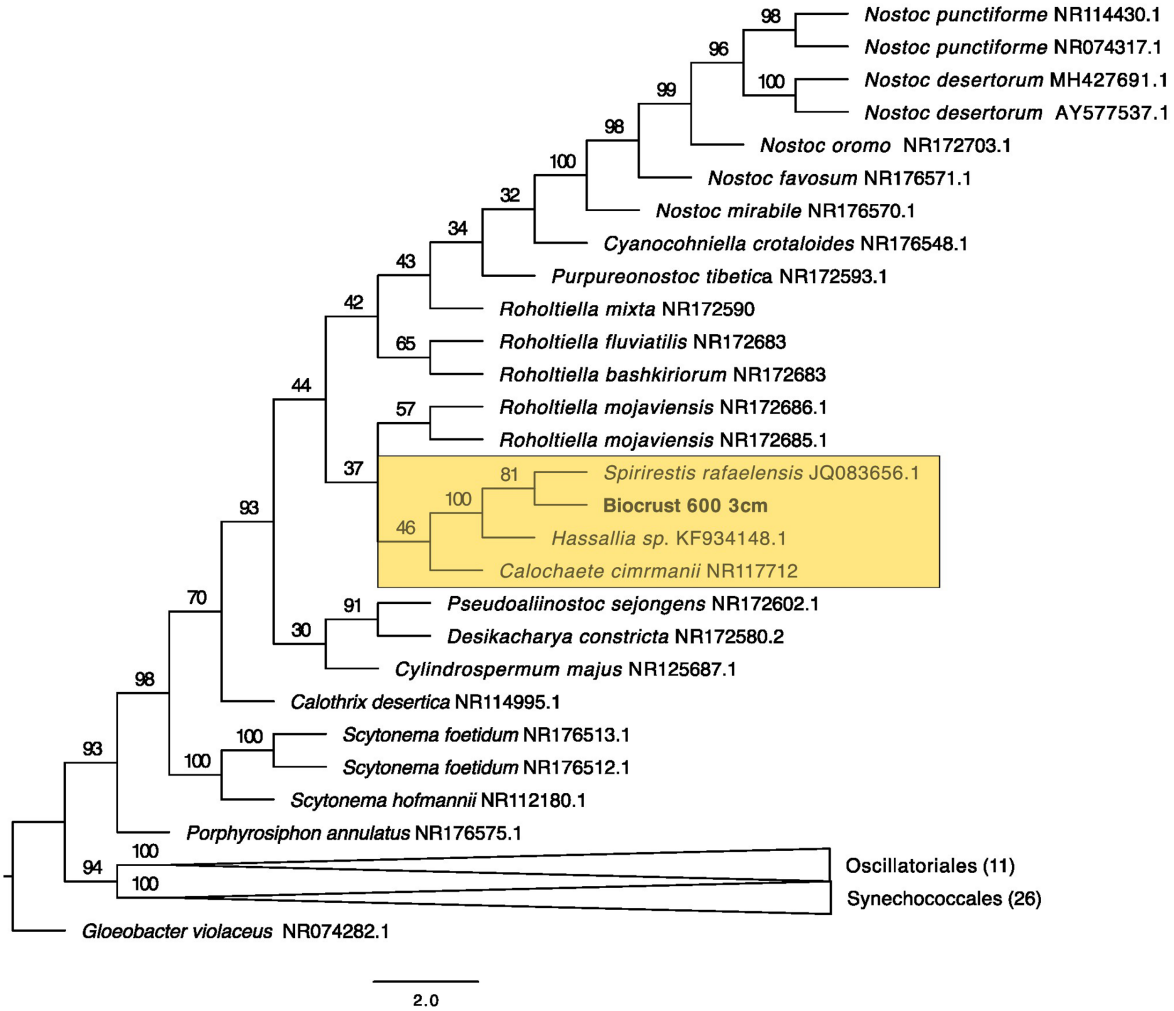
Maximum likelihood tree for Nostocales. Bootstrap values are denoted on the branches. Bootstrap values indicate the percent of the trees with that particular branch placement. Bootstrap values >0.70 are considered well supported.

**Figure 7:**
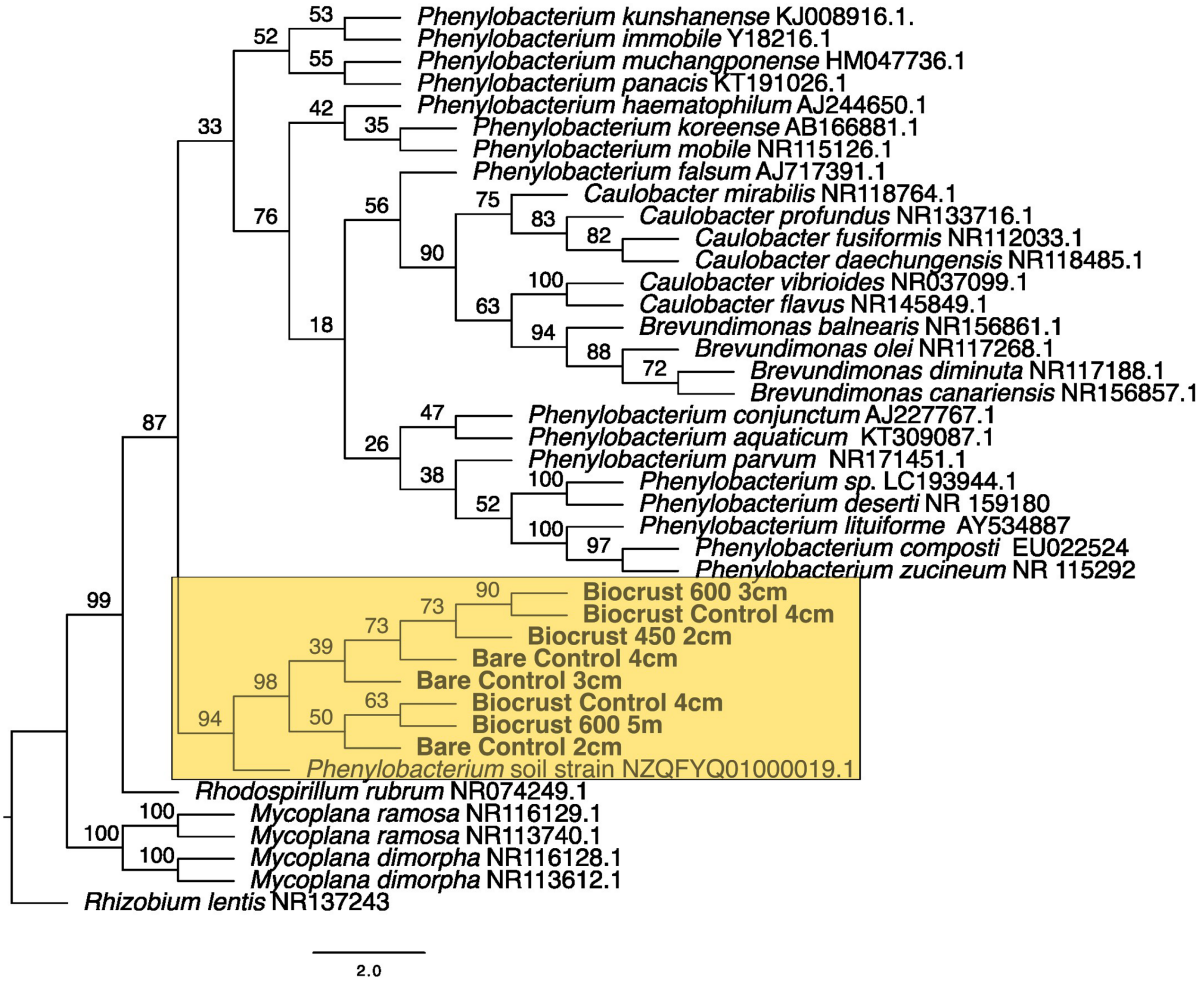
Maximum likelihood tree for Caulobacterales. Bootstrap values are denoted on the branches. Bootstrap values indicate the percent of the trees with that particular branch placement. Bootstrap values >0.70 are considered well supported.

**Figure 8:**
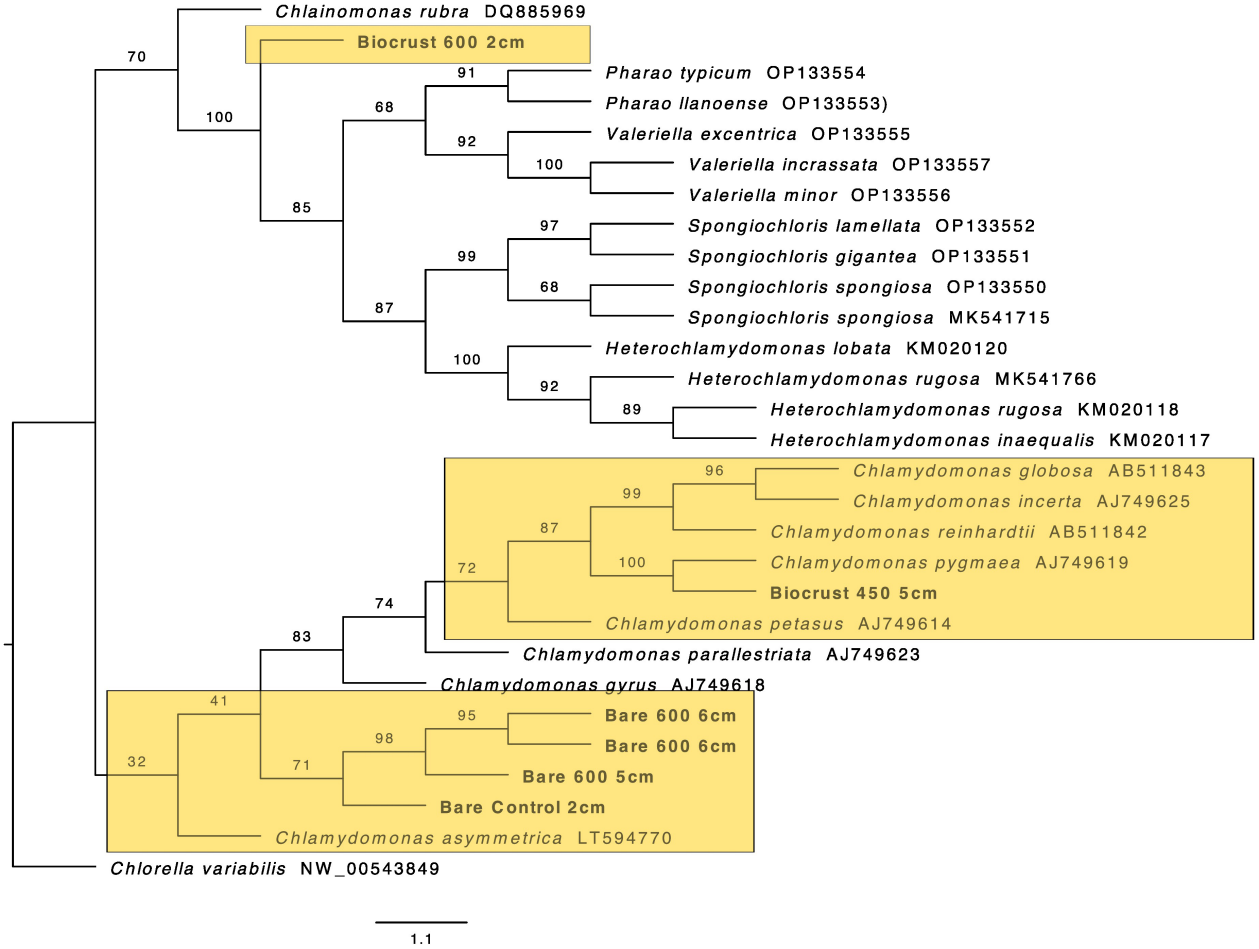
Maximum likelihood tree for Chlamydomonadales. Bootstrap values are denoted on the branches. Bootstrap values indicate the percent of the trees with that particular branch placement. Bootstrap values >0.70 are considered well supported.

The closest 16 s rRNA and ITS sequence matches are presented in Table 2. Nineteen sequences were obtained from bare soil and twelve from biocrust. Eleven were from the controls, fifteen were from the high temperature fire, and five were from the low temperature fire. Seven sequences were low quality and had ambiguous hits within the NCBI database, so these were not included in the phylogenetic trees. For bacteria the sequences spanned four orders: Oscillatoriales, Synechococcales, Nostocales, and Caulobacterales (Figures 4–7). The green algae sequences were in Chlamydomonadales (Figure 8).

**Table 2:** The first hit from the NCBI database for each culture.

NCBI Hit	Query cover	Per. Ident	Accession	Type	Temp	Depth
*Wilmottia murrayi* 23PC	92%	97.25%	KY288988.1	Bare	450	2
Uncultured fungus genomic DNA sequence	95%	90.05%	OU940418.1	Bare	450	6
Uncultured fungus genomic DNA sequence	95%	88.90%	OU940418.1	Bare	450	6
*Pseudophormidium americanum* KT18	66%	97.75%	MK861905.1	Bare	600	2
*Leptolyngbya* cf. *frigida* ACT684	96%	98.32	MK248006.1	Bare	600	4
*Trichocoleus desertorum*	93%	96.88%	MK478718.1	Bare	600	4
Uncultured Vahlkampfiidae	96%	90.42%	EU812478.1	Bare	600	4
*Spongiochloris spongiosa*	82%	94.71%	KM020025.1	Bare	600	5
Uncultured Chlamydomonadales isolate soil	24%	94.74%	MF482975.1	Bare	600	6
*Spongiochloris spongiosa*	60%	93.55%	KM020025.1	Bare	600	6
*Leptolyngbya* cf. *frigida* ACT684	93%	95.94%	MK248006.1	Bare	Control	1
*Brevundimonas* sp. LY-2	94%	97.32%	GU003879.1	Bare	Control	2
*Chlorococcum isabeliense*	70%	92.08%	LT594563.1	Bare	Control	2
*Brevundimonas* sp. MF30-B	92%	98.52%	CP038440.1	Bare	Control	3
*Brevundimonas* sp. MF30-B	93%	99.12%	CP038440.1	Bare	Control	4
*Leptolyngbya* cf. *frigida*	93%	97.69%	MK248006.1	Bare	Control	5
*Brevundimonas* sp. GENT16	74%	79.51%	LC094574.1	BSC	450	2
*Heterochlamydomonas* sp.	94%	99.53%	MT735195.1	BSC	450	5
Uncultured bacterium gene for 16S rRNA, partial sequence, clone: L1CLN37	95%	97.45%	AB696310.1	BSC	600	2
*Edaphochlorella mirabilis*	30%	94.90%	OM472009.1	BSC	600	2
*Tolypothrix* sp. Es_Yyy1600	90%	94.30%	KC463187.1	BSC	600	3
*Brevundimonas* sp. MF30-B	77%	78.68%	CP038440.1	BSC	600	3
*Brevundimonas* sp. FrW-Ado30	95%	98.16%	LC324780.1	BSC	600	5
*Phormidesmis* sp. WJT36-NPBG20	88%	96.55%	KJ939034.1	BSC	Control	4
*Brevundimonas* sp. SCU-B193	91%	84.98%	KJ000827.1	BSC	Control	4
*Brevundimonas* sp. FrW-Ado30	94%	98.83%	LC324780.1	BSC	Control	4

### 3.3. Isolates from 1 to 3 cm depth

We consider microbes living within the first 3 cm of the soil to be within the possible heat mortality zone during a fire based on the thermocouple data, our heat transfer model, and previous estimates of microbial thermotolerance (Kimura et al., 2015). Despite the high temperatures, we isolated several CFUs that were able to survive and grow after both fire temperatures.

At 2 cm depth from bare soil burned at 450°C, we isolated an organism closely related to *Wilmottia murrayi*. Also, at 2 cm depth, an isolate related to *Myxacorys californica* and a green alga isolate within the order Chlamydomoniales survived the 600°C fire (Figures 4, 5, and 8). Furthermore, a *Phenylobacterium* isolate from biocrust survived both fire temperatures at 2 and 3 cm depth (Figure 7). Additionally, an organism related to *Spirirestis rafaelensis* from 3 cm depth, also survived the 600°C fire (Figure 6).

### 3.4. Isolates from 4 to 6 cm depth

At 4–6 cm deep, the soil temperature was cooler, and the microorganisms here did not face the same heat stress as the shallower soil layers. A strain classifying to *Trichocoleus desertorum* was isolated from bare soil burned at 600°C at 4 cm deep (Figure 5). The *Phenylobacterium* isolate was also able to survive at 5 cm deep (Figure 7). Three isolates in a clade with *Phormidesmis* and four isolates in a clade with *Chlamydomonas* were found at 5 and 6 cm deep after both fire temperatures (Figures 5 and 8). Isolates related to *Trichocoleus desertorum* and *Drouetiella* were found at 6 cm depth following the 450°C and 600°C fires, respectively (Figure 5).

### 3.5. Isolates from controls

In addition to the burned soils, we also isolated microbes growing at different depths in unburned biocrust and bare soil cores. *Phenylobacterium* isolates were found at 2 cm, 3 cm, and 4 cm deep (Figure 7). A *Chlamydomonas* isolate was also found at 2 cm deep, and a *Phormidesmis* was isolated from 4 cm deep (Figures 5 and 8).

### 3.6. RNA results

We extracted RNA and transcribed it to cDNA. After sequencing the cDNA, we recovered 862 unique non-chimeric sequences from four biocrust samples. Three were burned at 600°C at 1, 2, and 8 cm, and one sample was from the control at 5 cm depth. We analyzed the dataset with both the Silva 138 database and the Cyanoseq database. Because of the limited sample size, we are unable to perform statistical analyses on these data. However, this does provide insight into which organisms are capable of surviving and responding rapidly after fire and merits future exploration.

**Figure 9:**
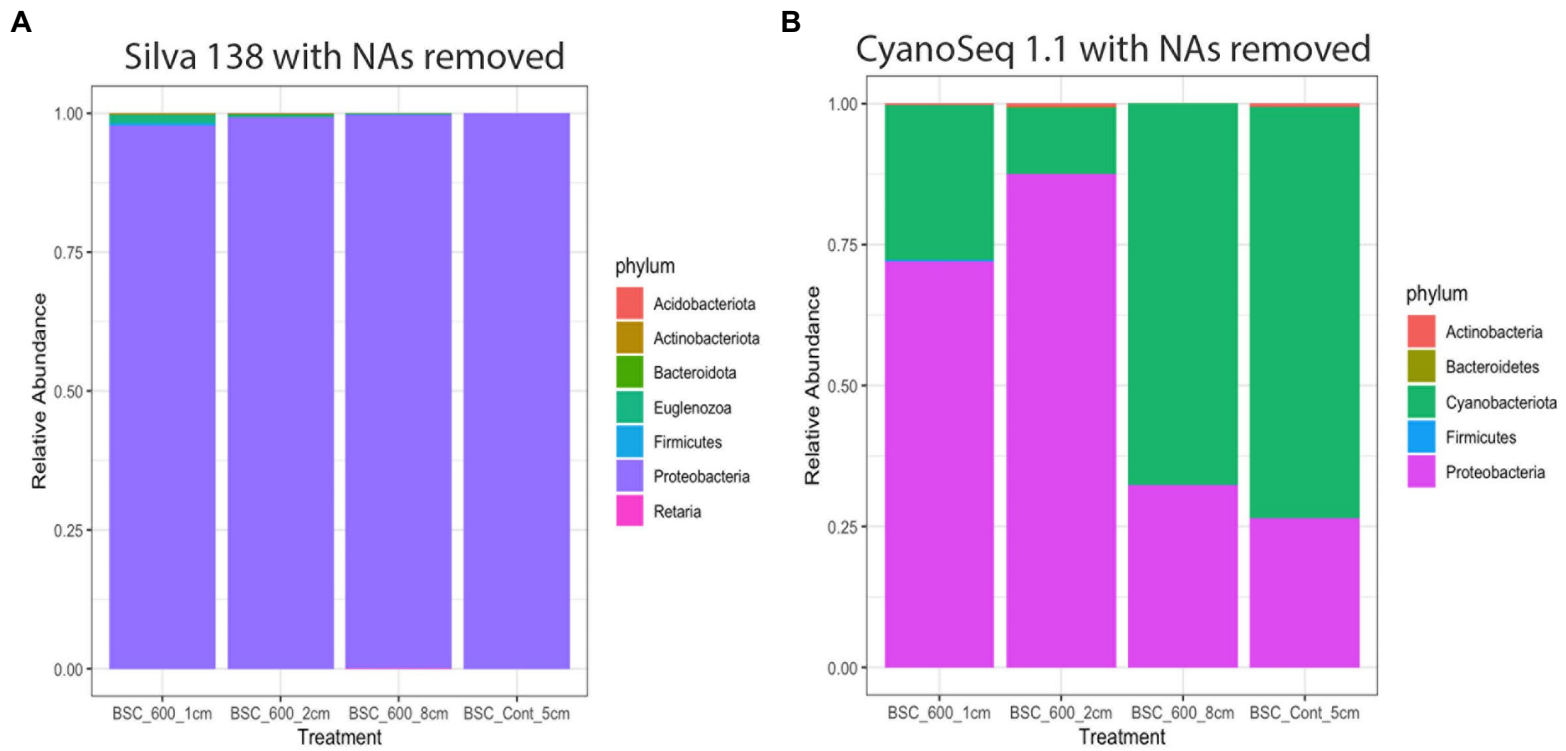
Relative abundance of each phylum after the unidentified sequences were removed based on the annotations from the Silva 138 database **(A)** and the CyanoSeq 1.1 database **(B)**.

The Silva database did not identify any Cyanobacteria sequences, rather it indicates that Proteobacteria is the most dominant phylum across all the sampled depths, though 83% of the sequences were unidentified at the phylum level (Table 3; Figure 9). Within Proteobacteria, Enterobacteriales (Gammaproteobacteria) was the dominant order and the other orders included Acetobacteriales, Burkholeriales, Pseudomonaldales, and Rickettsiales.

**Table 3:** The relative abundance of each phylum within each treatment based on the Silva database.

Silva database								
Treatment	Acidobacteriota	Actinobacteriota	Bacteroidota	Euglenozoa	Firmicutes	Proteobacteria	Retaria	NA
BSC_600_1cm	0	0.00040	0	0.0020	0.00060	0.14	0	0.86
BSC_600_2cm	0.00022	0.00022	0.00033	0.0013	0	0.30	0	0.69
BSC_600_8cm	0	0	0.00015	0	0.00015	0.15	0.000099	0.85
BSC_Cont_5cm	0	0	0	0	0	0.11	0	0.89

Using the CyanoSeq database resulted in fewer unidentified sequences. Sixty-five percent of the sequences were unidentifiable at the phylum level. There are fewer Cyanobacteria sequences in the top two centimeters of the soil (relative abundances of 0.052 and 0.041) compared to the deeper burned layer and the control (relative abundance 0.31 and 0.30). The control sample had the greatest relative abundance of Cyanobacteria ASVs (Table 4). Additionally, Proteobacteria were more dominant in the top centimeter of the soil (relative abundance of 0.14). Within Proteobacteria, the order Enterobacterales (Gammaproteobacteria) dominated all the treatments, including the control, suggesting possible contamination as Enterobacterales is a common contaminant though it may also be indicative of the r-selected nature of Gammaproteobacteria (Zhang et al., 2018). The other orders include Burkholderiales, Chromatiales, Hyphomicrobiales, and Rhodospirillales.

**Table 4:** The relative abundance of each phylum within each treatment based on the CyanoSeq database.

CyanoSeq database						
Treatment	Actinobacteria	Bacteroidetes	Cyanobacteriota	Firmicutes	Proteobacteria	NA
BSC_600_1cm	0.00040	0	0.052	0.00060	0.14	0.81
BSC_600_2cm	0.0021	0.00033	0.041	0	0.30	0.65
BSC_600_8cm	0	0.00015	0.31	0.00015	0.15	0.54
BSC_Cont_5cm	0.0024	0	0.30	0	0.11	0.59

In total, 74 ASVs within Cyanobacteriota were identified using the CyanoSeq database comprising 10,160 ASV copies. 30 ASVs were present in the sample burned at 1 cm deep, 14 ASVs at 2 cm deep, 29 ASVs at 8 cm deep, and 19 ASVs in the control. Twelve ASVs were shared among multiple treatments.

**Figure 10:**
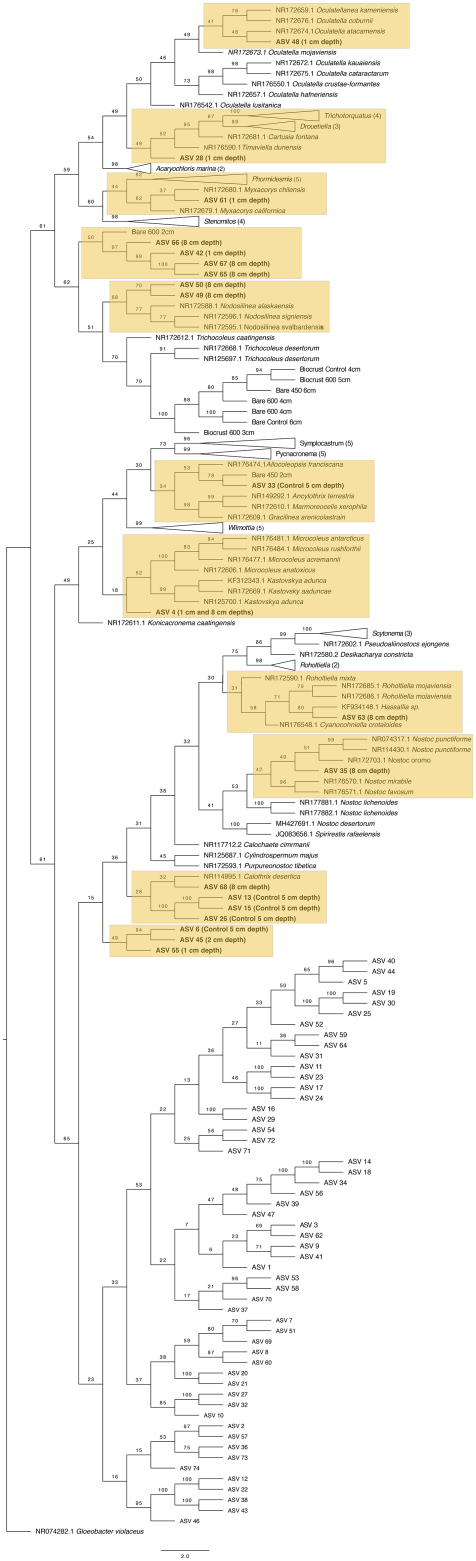
Maximum likelihood tree for 74 Cyanobacteriota ASVs with sequences from prokaryotic Cyanobacteria and the sequences derived from the 16S Sanger sequencing. Bootstrap values are denoted on the branches. Bootstrap values indicate the percent of the trees with that particular branch placement. Bootstrap values >0.70 are considered well supported. Labels refer to the treatment(s) where the ASVs were found. Only those well-placed within the tree indicate the treatment, for more information on the ASVs in each treatment, see Table 2.

**Figure 11:**
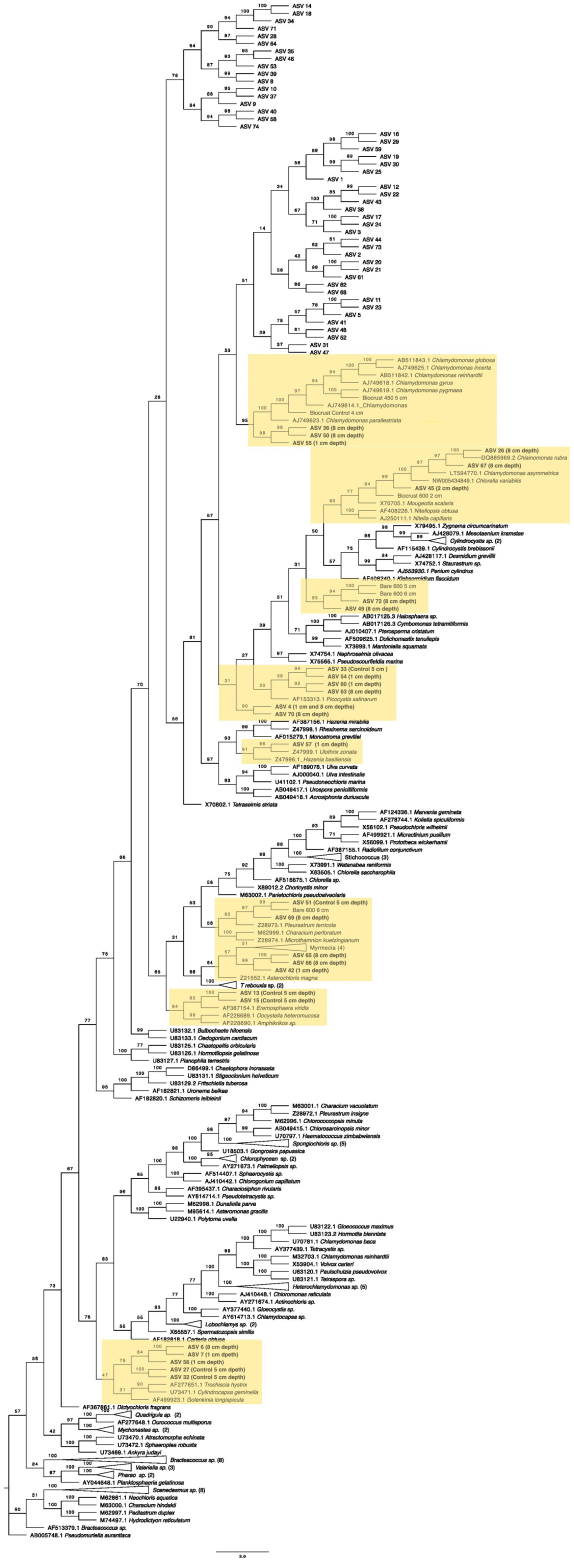
Maximum likelihood tree for 74 Cyanobacteriota ASVs with sequences from eukaryotic green algae and the sequences derived from the 16S Sanger sequencing. Bootstrap values are denoted on the branches. Bootstrap values indicate the percent of the trees with that particular branch placement. Bootstrap values >0.70 are considered well supported. Labels refer to the treatment(s) where the ASVs were found. Only those well-placed within the tree indicate the treatment, for more information on the ASVs in each treatment, see Table 2.

After extracting these sequences, we placed them within a phylogenetic tree containing the CyanoSeq ASVs, the Sanger sequences from this experiment, and representative sequences from NCBI. We created two phylogenetic trees – one composed of prokaryotic Cyanobacteria and one with eukaryotic algae using accession numbers described in Lewis and Lewis (2005) (Figures 10, 11). Of the 74 ASVs, 19 were well-situated within the Cyanobacteria phylogenetic tree and 27 were well-situated in the Eukaryotic algae tree. Fifteen ASVs were placed in both the prokaryotic and eukaryotic trees, indicating the difficulty in identifying Cyanobacteriota ASVs at even the domain level. For ASVs that were placed in both trees, we compared the bootstrap values based on the branch with the closest proposed relative. Of the ASVs that were placed in both trees, ASV 4, ASV 6, ASV 13, ASV 15, ASV 26, ASV 42, ASV 45, ASV 49, ASV 50, ASV 55, ASV 65, ASV 66, and ASV 67 were better placed within the eukaryotic algae tree. Only ASV 33 and ASV 63 were better placed in the prokaryotic tree (Supplementary Table 2).

Within the prokaryotic cyanobacteria tree (Figure 10), nine ASVs were placed within Synechococcales. ASV 48 was detected at 1 cm depth and was placed in a clade with the genus *Oculatella.* ASV 28 and ASV 61 were also found at 1 cm depth and are ambiguously placed within a clade with *Timaviella* and *Myxacorys*, respectively. ASVs 42, 65, 66, and 67 were also placed within Synechoccales but had more support in the eukaryotic tree. Within Oscillatoriales, ASV 33 from the control samples is in a clade with a Sanger sequence from bare soil burned at 450°C at 2 cm depth and with the genus *Allocoleopsis*, suggesting that the isolate we sequenced may also be found at greater depths in the control sample. Eight ASVs were placed within Nostocales with the majority (n = 6) of them detected either at 8 cm depth or in the control – although four had higher bootstrap values in the algae tree (ASV 6, ASV 13, ASV 15, and ASV 26). The ASVs well placed within Nostocales include ASV 63 in a clade with *Hassallia*, ASV 35 in a clade with *Nostoc* and ASV 68 in a clade with *Calothrix*.

Within the eukaryotic algae tree (Figure 11), three ASVs (ASV 36, ASV 50, and ASV 55) are in a clade with Chalmydomonas (bootstrap = 95) and with two of the Sanger sequences (Biocrust 450°C at 5 cm and Biocrust Control at 4 cm). Also, within the order Chalmydomonales, ASV 26 and ASV 67, both from 8 cm depth are in a clade with *Chlainomonas rubra*. ASV 45 from 2 cm depth, groups with the Chlamydomonas order and is similar to the Sanger sequence from Biocrust burned at 600°C at 2 cm depth – the same treatment that the ASV was found in. Furthermore, ASVS 49 and 72 both from 8 cm deep group with the Sanger sequences from 5 cm and 6 cm deep, also broadly within Chalmydomonales. ASV 4 and ASV 70 (from 1 cm and 8 cm depth) are also grouped within Chalmydomonales but the bootstrap value does not indicate strong support for this placement (bootstrap = 30). ASV 33 (control), ASV 54 (1 cm), ASV 60 (1 cm), and ASV 63 (8 cm) are grouped together and potentially related to *Picocystis* sp., though they are more similar to each other than to *Picocystis sp.,* and ASV 33 and ASV 63 had better support in the prokaryotic tree. However, ASV 57, found at 1 cm depth is in a supported clade with *Ulothrix zonata* and *Hazenia brasillensis*. The Sanger sequence from the bare soil and the 600°C fire at 6 cm depth is in a clade with ASV 51 and ASV 69 from the control and 8 cm deep, respectively. ASV 42, ASV 65, and ASV 66 are in a clade with *Myrmecia sp.* and *Asterochloris magna*. Furthermore, the ASVs 13 and 15 are in a sister clade with *Eremosphaera viridis.* Finally, within the class Chlorophyceae, five ASVs are in the same clade and are found at 1 cm (ASV 7 and ASV 56), 8 cm (ASV 6), and the control (ASV 27 and ASV32).

## 4. Discussion

Here we show evidence that microbes can survive a fire and grow, given the right conditions from 1–10 cm depth in the soil after an experimental fire. Based on the presence-absence of CFUs on the agar plates, we can conclude that both biofilm forming cyanobacteria and eukaryotic algae can survive fire. The sequences from these isolates reveal a mixed community of Cyanobacteria, Alphaproteobacteria and green algae. Although we had CFUs growing on a plate from the top centimeter of a burned biocrust sample, we were unsuccessful in extracting the DNA and sequencing the isolate and primarily focused the sequencing effort on the isolates from 2-6 cm deep. Unsuccessful DNA extractions may have occurred due to the complex nature of cyanobacterial cells which include surface structures like mucilaginous sheaths, slime, and capsules as well as a high polysaccharide content and secondary metabolites, all of which may inhibit DNA extraction (Singh et al., 2012).

Culturing experiments such as this do not account for the total biodiversity within soil as the majority of bacteria are not culturable (Vartoukian et al., 2010), and thus we are not capturing the diversity of organisms that survive and grow after fire at these depths. Our RNA analysis partially answers this question, but culturing and whole-community sequencing efforts must be combined to fully understand the effect of fire across the depth profile. Here, we highlight the isolates that were able to survive and grow after a fire and describe the potential adaptations or reasons for their success based on previously described relatives in the literature. These descriptions may be used as hypotheses for future experiments to directly test the mechanisms of heat tolerance and fire survival for these organisms.

### 4.1. 1–3 cm deep

We were able to sequence microbial isolates from 2 cm depth for both fire temperatures. A *Phenylobacterium* was found in biocrust and bare soils after both fire types, and was related to an isolate from soils with heavy metals (Li et al., 2019). *Phenylobacterium* is known to be abundant after fire. In an outdoor controlled fire, Lucas-Borja et al. (2019) found that *Phenylobacterium* was relatively more abundant in soils that were burned in a high severity fire (595°C) compared to the low severity fire (547°C) (Lucas-Borja et al., 2019). Additionally, Woolet and Whitman (2020) found that genera within *Phenylobacterium* increased in soil after fire. *Phenylobacterium* may be common in burned soils due to their ability to degrade aromatic compounds (Lingens et al., 1985). In a savanna ecosystem, *Phenylobacterium* OTUs were present in soils after the first rain following the fire season (de Souza and Procópio, 2022).

*Phenylobacterium* is ubiquitous among treatments as it is found in the bare control at 2, 3, and 4 cm, biocrust 600°C burn at 3 and 5 cm, and in the biocrust control at 4 cm. It was not found on the soil surface or in any of the burned bare treatments. Perhaps this is related to the lower temperatures we recorded with the thermocouples in the biocrust samples (Table 1) and the biocrusts protected *Phenylobacterium* from the heat at the shallower depths.

We also sequenced an alga isolate from 600°C at 2 cm using eukaryotic ITS primers and found that the strain was within the Chlamydomonadales order, though the lower taxonomic levels are ambiguous. Chlamydomonadales are prolific slime formers and develop flagellated cell stages (Turmel et al., 2008; Lemieux et al., 2015) – both traits could promote fire survival. Slime could act as a protective layer while flagella enable motility moving away from stressors. Further research is needed on how specifically cellular structures may be advantageous for thermal tolerance.

From the bare soil, the 450° fire isolate was similar in sequence to *Wilmottia murrayi* (bootstrap value = 74), a filamentous cyanobacteria in the Oscillatoriales order. *W. murrayi* has also been identified in freshwater systems in Antarctica (Strunecký et al., 2011). A similar species was found in biocrusts from a temperate desert in China (Wang et al., 2020). Previously, researchers have described *Wilmottia murrayi* as being found exclusively in cold and temperate regions of the world, although more recent evidence suggest it has a wider distribution (Machado-De-Lima et al., 2017), and perhaps a wider range of thermotolerance. Cyanobacteria within Oscillatoriales form distinct exopolysaccharide sheaths around their cell walls and are able to glide within the soil (Garcia-Pichel and Pringault, 2001). These two traits have been hypothesized to promote desiccation tolerance (Garcia-Pichel and Pringault, 2001) but may also function as a mechanism to protect against fire impacts.

The Nostocales we isolated from 600°C at 3 cm depth was placed within a clade with *Spirirestis rafaelensis*. The estimate from our model predicts that these species experienced temperatures >100°C. *Spirirestis* is known from arid soils and from biocrusts, however, records are sparse and the data restricted to undisturbed and well developed biocrusted soils, and it has been speculated that this species may be rare (Baldarelli et al., 2022). It can form firm exopolysaccharide sheaths as well as heterocysts which may be helpful for survival under extreme temperatures (Flechtner et al., 2002).

We isolated a Synechococcales strain from bare soil burned at 600°C that is closely related to *Myxacorys* which is filamentous and has been isolated from biocrusts in North and South America (Pietrasiak et al., 2019). Found predominantly in deserts, this genus develops distinct slime caps and soft exopolysaccharide sheath (Pietrasiak et al., 2019) which may represent specific a specific adaptation allowing it to cope with the intense heat from wildland fires.

### 4.2. 4–6 cm deep

The soil from 4 to 6 cm deep experienced a broad temperature range from approximately 50–100°C. At these depths we isolated organisms grouped with *Trichocoleus* sp., *Phormidesmis* sp., *Drouetiella* sp., *Phenolobacterium* sp., and *Chlamydomonas* sp. *Trichocoleus desertorum* is common in dryland soils and biocrusts (Mühlsteinova et al., 2014) and is characterized by gliding motility and copious exopolysaccharide sheath development similar to *Wilmotia* although it belongs to the order Synechococcales. *Trichocoleus* isolates have been only previously found in unburned soils during an assessment of biocrust recolonization after a fire in a juniper woodland (Warren et al., 2015), suggesting that this genus may not be fire resistant. However, our data suggests it may be able to survive if it resides at deeper soil levels. *Phormidesmis* is common in soils and appears to be heat sensitive (Chrismas et al., 2015; Raabova et al., 2019). The genus *Phormidesmis*, is diverse and found across a variety of habitats, suggesting that this genus may have varying thermal tolerances, but our study suggested it was not as thermotolerant as the other isolates. *Drouetiella* sp. are filamentous cyanobacteria, and the genus contains three named species and has been isolated from Antarctica, from a seep wall in the Colorado Plateau, and from tropical stagnant water in Brazil (Mai et al., 2018), suggesting a wide range of thermotolerance.

The presence of *Phenolobacterium* in both the surface layers and the deeper soil layers suggests that this isolate is ubiquitous across the soil profile and has a broad thermal tolerance. Four algal isolates from 4 to 6 cm depth closely match with *Chlamydomonas*, though the algal isolate that survived at 2 cm depth is in a separate clade. This suggests a difference in thermal tolerance between isolates within this order.

### 4.3. Control isolates

Interestingly, we were unable to successfully culture and sequence control isolates from Nostocales and Oscillatoriales, though of course, this does not mean that organisms from these orders were not present. Rather, the methodology of single culture isolation biased the sequences we were able to analyze. Within Synechococcales, we isolated organisms from the control soils and biocrusts from 4 cm depth that were in a clade with *Phormidesmis nigrescens*. *Phenylobacterium* was also present in the control samples from 2 cm – 4 cm depth. For the green algal isolates, the control from 2 cm depth was placed in a similar clade with isolates from 600°C at 6 cm, supporting the hypothesis that *Chlamydomonas* are less thermal tolerant and have the potential to live closer to the soil surface during average conditions.

### 4.4. Bare versus biocrust soil

The only Oscillatoriales isolate was found in bare soil while the only Nostocales isolate was found in the biocrust. Both bare and biocrust isolates were represented in the other orders. The biocrust and bare samples were also collected within 1 meter of each other in an area with a patchy distribution of biocrusts and bare ground. Here we found cyanobacteria genera common to both biocrust and soils in the bare treatment. Therefore, regardless of the formation of a biocrust, the microbial communities, especially below the first centimeter may be similar. Previous research at SMER identified common biocrust cyanobacterial genera such as *Microcoleus, Anabaena,* and *Nostoc* in bulk soil samples at the same site where collection occurred for this experiment (Pérez Castro et al., 2019), though an assessment of the SMER biocrust microbial community is needed.

We also had more success extracting the DNA and performing PCR with the samples collected from the bare soils, biasing our results toward a more complete picture of the bare soils. This may be due to the abundance of secondary metabolites often found in biocrusts which can interfere with DNA extraction (Margolis et al., 2022). Future research should use more robust DNA extraction and sequencing methods to understand which microbes are found after fire at each depth.

Despite there being mixed cultures growing on the plates, only unialgal isolates were used for DNA extraction and subsequent sequencing. We therefore think there was likely a mix of Cyanobacteria and Alphaproteobacteria in both cover types. Additionally, the PCR and sequencing was unsuccessful for several of our isolates due to the contamination or the presence of a mixed culture. Attempting to classify cyanobacteria through mixed cultures leads to a risk of misidentification (Anagnostidis and Komarek, 1999). We took care to isolate individual cultures by transferring them to their own BG-11 plate where they were allowed to grow alone, but contamination still could have occurred.

Based on the thermocouple data, the biocrust treatments had lower temperatures at each depth, which may have altered the types of microbes we were able to culture. However, this result should not be taken at face value. The placement of the thermocouples was haphazard. We used a ruler to place the thermocouples at the exact locations prior to each burn, but they could have shifted as we moved the mug into the pyrocosm. This may also explain why the thermocouples are about a centimeter off from the values predicted by our model. The purpose of this experiment was not to test if biocrusts maintained cooler temperatures in deeper soil layers during a fire, but based on these results, future research should examine this hypothesis with most predictable and accurate measurements at each depth.

### 4.5. RNA sequencing

Both the CyanoSeq and SILVA databases were unable to classify the majority of the organisms within the cDNA samples, however the CyanoSeq database did classify the samples with fewer NAs and identified more Cyanobacteria sequences. Gammaproteobacteria was the dominant class in both databases and primarily consisted of ASVs within Enterobacteriales. Gram-negative bacteria are generally regarded as more opportunistic and can grow rapidly after a disturbance.

Gammaproteobacteria are often considered to be r-selected (Zhang et al., 2018). It is not surprising that fast-growing bacteria with large numbers of ribosomes per cell would dominate the rRNA content of soil after a disturbance (Bremer and Dennis, 2008). However, the two databases differed in the bacterial orders they classified, perhaps because CyanoSeq performs better for Cyanobacteria sequences while Silva is preferred for other bacterial phyla.

Based on the ASVs we extracted from CyanoSeq, there are several cyanobacteria and algae ASVs that are active at various depths after a fire, including the top centimeter of the soil and biocrust, though more sampling is needed to identify the specific organisms active after a fire at each depth. Interestingly, several ASVs from both 8 cm and 1 cm depth were placed in a clade with a Sanger sequence from our culturing efforts burned at 600°C at 2 cm depth—although these 4 ASVs were better placed within the eukaryotic tree. ASV 61 from 1 cm depth was placed in a clade with *Myxacorys* (bootstrap value = 62), similar to the Bare 600°C at 2 cm Sanger sequence, suggesting that ASVs similar to *Myxacorys* are ubiquitous at the soil surface following a fire. Furthermore, ASV 33 isolated from the control sample was grouped with our Sanger sequence from Oscillatoriales from 2 cm depth burned at 450°C (bootstrap value = 78) – indicating cohesion between our culturing efforts and sequencing. The Sanger sequence was initially placed in a clade with *Wilmottia* and when it was included in a tree with all the ASVs, it is in a clade with *Allocoleopsis*. The movement of the Sanger sequence between clades suggests that we do not fully understand the identity of this organism. In culture, *Allocoleopsis* was unable to grow at 45°C, though it was not correlated with environmental variables such as mean annual temperature and mean annual precipitation in field studies (Moreira et al., 2021). It is not common in hot deserts but is frequently found in biocrusts (Moreira et al., 2021). Results from this study suggest that a *Allocoleopsis* or *Wilmottia-*like organism is capable of withstanding fire near the soil surface.

There were also differences in depth stratification between the ASVs. The ASVs within Synechococcales were found at both 1 cm and 8 cm depths – the clades with *Oculatella*, and *Timaviella* harbored ASVs from the top centimeter while *Nodosilinea* contained two ASVs found only at 8 cm depth – though the ASVs grouped with *Nodosilinea* had higher bootstrapped values in the eukaryotic tree. The genus *Oculatella* is widely distributed in both arid and temperate environments, often in biocrusts (Becerra-Absalón et al., 2020). Previous studies have isolated *Oculatella* from thermal springs above 50°C (Brenes-Guillén et al., 2021) which suggests this genera is thermotolerant, and may respond positively to fire as indicated by the ASVs detected in the present study. The genus *Timaviella* is also in a clade with *Trichotorquatus*. One of the type species for the genera, *Trichotorquatus maritimus*, was isolated from the biocrust at SMER to understand biocrust cultivation and restoration (Pietrasiak et al., 2021; Reeve et al., 2023). The ASV detected in this study may be related to *Trichotorquatus maritumus* and may also show rapid growth on native soils (Reeve et al., 2023).

The ASVs well-placed within Nostocales include ASVs grouping with *Hassallia, Nostoc*, and *Calothrix* —all from 8 cm depth. This suggests that some strains within Nostocales are less heat tolerant and are perhaps more abundant at deeper soil depths.

Within the eukaryotic tree, there were several green algal strains found on the soil surface and in the deeper soil depths. In total, 13 ASVs were found either at 8 cm deep or in the controls, and 6 ASVs were found at 1 or 2 cm deep. Similar to the Sanger sequences, the majority of the ASVs were found in the Chlamydomonas order. Within the genus *Chlamydomonas*, two of the Sanger sequences from the control and 5 cm deep were in a clade with ASVs from 8 cm and one ASV from 1 cm, suggesting that *Chlamydomonas* is abundant at lower depths, but perhaps some strains can survive the heat from the fire on the surface. This genus has frequently been studied to understand heat shock. The heat shock protein genes are expressed at temperatures around 40°C (Schroda et al., 2015), which suggests it may have mechanisms to survive thermal stress, but more research is needed to understand the upper range of thermal tolerance within soil. Furthermore, two ASVs from 8 cm deep grouped with *Chlainomonas* which is a genus known for being cold-tolerant (Hoham and Remias, 2020), perhaps explaining why these ASVs are found in the deeper soil, where the temperatures were cooler. One ASV from 2 cm deep grouped with our Sanger sequence, also from 2 cm deep, likely related to *Chlorella*. Some *Chlorella* strains have been isolated from hot springs and grow in culture up to 45°C (Sakai et al., 1995; Ma et al., 2015), and thus may have adaptations for fire-derived heat stress. ASV 57 was also found near the soil surface and is in a clade with *Ulothrix.* In a study assessing the recolonization of burned soil, *Ulothrix* recolonized the soil in the top centimeter, which is supported by the presence of ASV 57 (Myers et al., 2003). Additionally, ASV 42 was found at 1 cm and is in a clade with *Myrmecia*, a common green alga in biocrusts (Rosentreter and Belnap, 2001). ASV 7 and ASV 56, also from 1 cm deep, are closely related to ASV 6 from 8 cm and ASVs 27 and 32 from the control. These ASVs are all placed within a clade with genera in the class Chlorophyceae; which based on these data, exhibits a range of thermotolerances. The other ASVs from 1 cm deep, ASV 4, ASV 54, and ASV 60, are ambiguously defined within Chlamydomonas.

Combined, the two maximum-likelihood trees for prokaryotic and eukaryotic Cyanobacterioda show a diversity of organisms at each depth. The strains similar to the genera *Oculatellanea, Timaviella Myxacorys, Chlamymomonas,* and *Ulothrix* may be more tolerant of the high temperatures on the soil surface during a fire and are able to swiftly recover, while stains similar to *Hassallia*, *Nostoc, Calothrix,* and *Chlainomonas* prefer cooler soil temperatures.

Despite the limited sample size, the RNA sequencing provided valuable information about the soil microorganisms that are able to survive a fire. By using RNA, we were able to determine which types of organisms are metabolically active after a fire. This proof-of-concept experiment showed that opportunistic Gammaproteobacteria are abundant after a fire, and some Cyanobacteria are also able to withstand the heat from the fire and recover quickly. The ASVs placed within the prokaryotic cyanobacteria and eukaryotic green algal tree provide more information about the types of organisms that are found at each depth after a fire. Furthermore, several of the ASVs were in clades with our Sanger sequences which leads to cohesion between the two parts of this study.

This study suggests that there are multiple ways for biocrust and bare soil microbial communities to recover after a fire. We showed that organisms are sheltered from the heat from the fire in deeper soil layers, and these organisms may be able to recolonize the soil and reform a biocrust after a fire. With culturing and RNA sequencing, we also showed that several organisms are able to survive the heat of the fire at the soil surface and 2 cm below the soil surface. These organisms may be instrumental in the early successional processes of reforming biocrusts and shaping the soil microbial community after a fire.

## 5. Conclusion

This is a step toward understanding how biocrust and soil microbes respond to fire. We showed that culturable microbes can grow from 2 to 10 cm deep after a fire. We also found that Gammaproteobacteria are the dominant group active after a fire, though some cyanobacteria ASVs are also present – even on the soil surface. Furthermore, we showed that a simulated fire with a blowtorch can be used to answer small scale questions related to how organisms respond to fire. The method can be modified for future studies to fit research needs.

## Data availability statement

The datasets presented in this study can be found in online repositories. The names of the repository/repositories and accession number(s) can be found at: NCBI - https://github.com/briannepalmer/Biocrust-Burn-Experiment.

## Author contributions

BP and DL designed the study. BP and PC performed the laboratory analyses. BP and NP created the phylogenetic trees. BP wrote the first draft of the manuscript. DL, NP, and PC edited the manuscript. All authors contributed to the manuscript revision, read, and approved the submitted version.

## Funding

Funding for the research was provided by the Joint Doctoral Program in Ecology at San Diego State University, Department of Biology. Additional funding for publication costs was provided by NSF #2154746 (RAPID: Interactive effects of wildfire and severe drought on plants, soil microbes and C storage in a semiarid shrubland ecosystem, PI: Lipson, co-PI’s: Xu, Cleland).

## Conflict of interest

The authors declare that the research was conducted in the absence of any commercial or financial relationships that could be construed as a potential conflict of interest.

## Publisher’s note

All claims expressed in this article are solely those of the authors and do not necessarily represent those of their affiliated organizations, or those of the publisher, the editors and the reviewers. Any product that may be evaluated in this article, or claim that may be made by its manufacturer, is not guaranteed or endorsed by the publisher.
