# Supplementary material for: Using simulated wildland fire to assess microbial survival at multiple depths from biocrust and bare soils
Source: Front Microbiol. 2023 Mar 17;14:1123790. doi: 10.3389/fmicb.2023.1123790 (PMC10064808; doi:10.3389/fmicb.2023.1123790)
Supplement: Supplementary file 1 [file Table_1.DOCX]

Supplementary Table 1: A list of the NCBI accession numbers to create the maximum-likelihood trees in Figures 4-8.

| **Alphaproteobacteria** | **Chlamydomonales** | **Nostocales** | **Oscillatoriales** | **Synechoccales** |
| --- | --- | --- | --- | --- |
| AB166881.1 | DQ885969.2 | NR_114430.1 | NR_172707.1 | KF417430.1 |
| AJ227767.1 | KM020118.1 | NR_74317.1 | NR_172706.1 | KU219715.1 |
| AJ244650.1 | MK541766.1 | MH427691.1 | NR_172704.1 | KU219737.1 |
| AJ717391.1 | KM020120.1 | AY577537.1 | NR_172705.1 | KU219739.1 |
| AY534887.1 | MK541715.1 | NR_172703.1 | NR_177880.1 | KU219740.1 |
| EU022524.1 | OP133550.1 | NR_176571.1 | NR_177881.1 | NR_074282.1 |
| HM047736.1 | OP133551.1 | NR_176570.1 | NR_177882.1 | NR_125697.1 |
| KJ008916.1 | OP133552.1 | NR_176548.1 | NR_176484.1 | NR_172564.1 |
| KT191026.1 | OP133553.1 | NR_172593.1 | NR_176477.1 | NR_172587.1 |
| KT309087.1 | OP133554.1 | NR_172590.1 | LR655686.1 | NR_172588.1 |
| LC193944.1 | OP133555.1 | NR_172684.1 | LR655681.1 | NR_172591.1 |
| NR_37099.1 | OP133556.1 | NR_172683.1 | NR_176481.1 | NR_172594.1 |
| NR_74249.1 | OP133557.1 | JQ083656.1 | NR_172606.1 | NR_172595.1 |
| NR_112033.1 | LT594770.1 | KF934148.1 | NR_149292.1 | NR_172596.1 |
| NR_113612.1 | AB511842.1 | NR_172686.1 | NR_172610.1 | NR_172612.1 |
| NR_113740.1 | AB511843.1 | NR_172685.1 | MF581661.1 | NR_172657.1 |
| NR_115126.1 | AJ749625.1 | NR_172602.1 | NR_151862.1 | NR_172659.1 |
| NR_115292.1 | AJ749619.1 | NR_172580.2 | NR_172671.1 | NR_172662.1 |
| NR_116128.1 | NW_5434849.1 | NR_125687.1 | KF312350.1 | NR_172663.1 |
| NR_116129.1 | AJ749614.1 | NR_114995.1 | NR_172670.1 | NR_172668.1 |
| NR_117188.1 | AJ749623.1 | NR_176513.1 | KF312349.1 | NR_172672.1 |
| NR_117268.1 | AJ749618.1 | NR_176512.1 | NR_172669.1 | NR_172673.1 |
| NR_118485.1 |  | NR_112180.1 | NR_125700.1 | NR_172674.1 |
| NR_118764.1 |  | NR_176575.1 | KF312343.1 | NR_172675.1 |
| NR_133716.1 |  | NR_176481.1 | NR_172611.1 | NR_172676.1 |
| NR_137243.1 |  | NR_176477.1 | NR_172608.1 | NR_172679.1 |
| NR_145849.1 |  | NR_115154.1 | NR_172607.1 | NR_172680.1 |
| NR_156857.1 |  | NR_112154.1 | MF581663.1 | NR_172681.1 |
| NR_156861.1 |  | NR_074282.1 | MF581659.1 | NR_172693.1 |
| NR_159180.1 |  |  | NR_172609.1 | NR_172704.1 |
| NR_171451.1 |  |  | NR_172658.1 | NR_172705.1 |
| NZ_QFYQ01000019.1 |  |  | EF654065.1 | NR_172706.1 |
| Y18216.1 |  |  | NR_172594.1 | NR_172707.1 |
