# Supplementary material for: Using simulated wildland fire to assess microbial survival at multiple depths from biocrust and bare soils
Source: Front Microbiol. 2023 Mar 17;14:1123790. doi: 10.3389/fmicb.2023.1123790 (PMC10064808; doi:10.3389/fmicb.2023.1123790)
Supplement: Supplementary file 2 [file Table_2.DOCX]

Supplementary Table 2: The number of copies of each Cyanobacteriota ASV found in each treatment. As well as, the bootstrap value from the eukaryotic and/or prokaryotic maximum-likelihood trees. The bootstrap values are derieved from the branch with the closest defined relative (either an NCBI or Sanger sequence). * indicate which tree (prokaryotic or eukaryotic) had the greatest bootstrap support for that ASV.

| **ASV** | **Number of Copies** | **Treatment** | **Eukaryotic Tree Booststrap Values** | **Prokaryotic Tree Bootstrap Values** |
| --- | --- | --- | --- | --- |
| ASV01 | 2759 | Control | - | - |
| ASV02 | 1591 | Control | - | - |
| ASV03 | 1579 | Control | - | - |
| ASV04 | 16 | 1cm | 90* | 18 |
| ASV04 | 1357 | 8cm | 90* | 18 |
| ASV05 | 1297 | Control | - | - |
| ASV06 | 1262 | 8cm | 47* | 15 |
| ASV07 | 17 | 1cm | 47 | - |
| ASV07 | 1182 | 8cm | - | - |
| ASV08 | 8 | 1cm | - | - |
| ASV08 | 3 | 2cm | - | - |
| ASV08 | 908 | 8cm | - | - |
| ASV09 | 9 | 1cm | - | - |
| ASV09 | 873 | 8cm | - | - |
| ASV10 | 12 | 1cm | - | - |
| ASV10 | 17 | 2cm | - | - |
| ASV10 | 324 | 8cm | - | - |
| ASV10 | 11 | Control | - | - |
| ASV11 | 53 | 1cm | - | - |
| ASV11 | 45 | 8cm | - | - |
| ASV11 | 63 | Control | - | - |
| ASV12 | 48 | 1cm | - | - |
| ASV12 | 25 | 8cm | - | - |
| ASV12 | 47 | Control | - | - |
| ASV13 | 84 | Control | 83* | 28 |
| ASV14 | 43 | 2cm | - | - |
| ASV14 | 29 | 8cm | - | - |
| ASV15 | 67 | Control | 83* | 28 |
| ASV16 | 10 | 1cm | - | - |
| ASV16 | 10 | 8cm | - | - |
| ASV16 | 41 | Control | - | - |
| ASV17 | 18 | 1cm | - | - |
| ASV17 | 36 | Control | - | - |
| ASV18 | 52 | Control | - | - |
| ASV19 | 30 | 2cm | - | - |
| ASV19 | 19 | 8cm | - | - |
| ASV20 | 37 | 1cm | - | - |
| ASV21 | 28 | 1cm | - | - |
| ASV22 | 27 | 2cm | - | - |
| ASV23 | 27 | 2cm | - | - |
| ASV24 | 7 | 2cm | - | - |
| ASV24 | 13 | 8cm | - | - |
| ASV25 | 18 | 1cm | - | - |
| ASV26 | 18 | 8cm | 100* | 28 |
| ASV27 | 17 | Control | 47 | - |
| ASV28 | 15 | 1cm | - | 49 |
| ASV29 | 13 | 2cm | - | - |
| ASV30 | 13 | 1cm | - | - |
| ASV31 | 13 | 1cm | - | - |
| ASV32 | 13 | Control | 47 | - |
| ASV33 | 13 | Control | 20 | 78* |
| ASV34 | 12 | 1cm | - | - |
| ASV35 | 11 | 8cm | - | 49 |
| ASV36 | 11 | 8cm | 95 | - |
| ASV37 | 8 | 1cm | - | - |
| ASV38 | 8 | Control | - | - |
| ASV39 | 7 | 2cm | - | - |
| ASV40 | 5 | 2cm | - | - |
| ASV41 | 5 | 1cm | - | - |
| ASV42 | 4 | 1cm | 57* | 50 |
| ASV43 | 4 | 8cm | - | - |
| ASV44 | 4 | Control | - | - |
| ASV45 | 3 | 2cm | 98* | 15 |
| ASV46 | 3 | 2cm | - | - |
| ASV47 | 3 | 1cm | - | - |
| ASV48 | 3 | 1cm | - | 48 |
| ASV49 | 3 | 8cm | 95* | 68 |
| ASV50 | 3 | 8cm | 95* | 68 |
| ASV51 | 3 | Control | 99 | - |
| ASV52 | 2 | 2cm | - | - |
| ASV53 | 2 | 2cm | - | - |
| ASV54 | 2 | 1cm | 20 | - |
| ASV55 | 2 | 1cm | 95* | 15 |
| ASV56 | 2 | 1cm | 47 | - |
| ASV57 | 2 | 1cm | 66 | - |
| ASV58 | 2 | 1cm | - | - |
| ASV59 | 2 | 1cm | - | - |
| ASV60 | 2 | 1cm | 20 | - |
| ASV61 | 2 | 1cm | - | 37 |
| ASV62 | 2 | 1cm | - | - |
| ASV63 | 2 | 8cm | 20 | 80* |
| ASV64 | 2 | 8cm | - | - |
| ASV65 | 2 | 8cm | 57* | 50 |
| ASV66 | 2 | 8cm | 57* | 50 |
| ASV67 | 2 | 8cm | 97* | 50 |
| ASV68 | 2 | 8cm | - | 32 |
| ASV69 | 2 | 8cm | 87 | - |
| ASV70 | 2 | 8cm | 90 | - |
| ASV71 | 2 | 8cm | - | - |
| ASV72 | 2 | 8cm | 94 | - |
| ASV73 | 2 | 8cm | - | - |
| ASV74 | 2 | Control | - | - |
